# Medical Commentaries on Puerperal Fever, Vermination, and Water in the Head

**Published:** 1836-10

**Authors:** 


Art. III.—*Medical Commentaries on Puerperal Fever, Vermination, and Water in the Head.* By John Alexander, m.d., Medical Officer to the General Dispensary for Children, and Bank of Manchester, &c.—*London*, 1836. 8vo. pp. 70.

Of the many minor defects and errors which constantly meet our eye in the actual condition of the profession in this country, there is no one which seems to us more to claim reprehension than the desire so prevalent among our younger brethren to come forth as authors, before experience and extensive observation have enabled them to obtain a sufficient stock of that superior knowledge which should seem to be an essential qualification of all who profess to instruct others. Not that we would regard with reproving or even cold looks the efforts of youthful genius or industrious research, but, on the contrary, count it as one of our dearest duties to foster and promote every thing of the kind. For such juvenile essays, however, the numerous journals which admit of what is termed Original Communications are the appropriate receptacles; as the simple record of facts is the most becoming occupation of the pens of those who can only be considered in the condition of learners, and not of teachers. Whenever we see any gentleman of this class coming forward as a public instructor,—that is, as the author of a book on a practical subject,—we experience sincere regret, because such publications are (with rare exceptions) generally the source of future regret to their authors, when longer experience has proved to them that, when they sought to teach, they stood most in need of learning. Such were our feelings on reading the present pamphlet; and such, if we are not very much mistaken, will hereafter be those of its respectable but youthful author.

Of the three essays of which this pamphlet consists, the last two, on Vermination and Hydrocephalus, do not contain a single observation offering the least claim or pretence to novelty, while they display numerous omissions of importance, and not a few positive errors.

The Essay on Puerperal Fever lays direct claim to novelty, being characterized by its author as containing "doctrines professedly peculiar;" but its sole merit consists in urging on the inexperienced practitioner (no man of experience can stand in need of such a warning,) the necessity of carefully discriminating the varieties and stages of the different morbid affections commonly classed under the general name of Puerperal Fever. As to the alleged *peculiarity* of the doctrines relative to the pathology of the disease, we believe that those who have reflected on that condition of the nervous system which often succeeds to sudden or severe shocks or impressions of any kind, and which we have pretty fully discussed in our last Number, (see the review of Mr. Travers's work on Constitutional Irritation,) will have nothing to learn from Dr. Alexander's "doctrines." What he calls exclusively puerperal fever is either the variety particularly adverted to in the preceding notice of Mr. Moore's work, or a particular stage of some of the other varieties; the form or stage, namely, of irritation or depression of vital power, of the existence and general character of which no practitioner of experience can be ignorant. And, as to the degree of additional light thrown on its intimate pathology by our author, we leave the reader to judge from Dr. A.'s own definition of the disease.

"I conceive it [puerperal fever] to be *a depressing affection of the abdominal nerves,* (as well as, in less degree, of the nervous system generally,) *strictly sui generis*; of which, from the peculiar nature of their functions, we are unable to take any physical cognizance, beyond such as relates to the observance of their morbid influences on the human frame. I apprehend it to be perfectly distinct from an inflammatory affection, although frequently terminating in or leading to one." (P. 12.)

No one will dissent from Dr. Alexander's opinion, that the morbid state which characterizes the first stage of many cases of puerperal fever, and the whole progress of some, (as, for instance, of that form which, in the preceding article, we have termed "the real and genuine puerperal fever,") is different from inflammation, or is, at least, (to avoid all possible misapprehension,) inflammation so modified by antecedent conditions of a different kind as not to bear the usual treatment of inflammation; and we are surprised to find him stating that such a state is "almost universally reported" to be inflammation. Of the frequent coexistence of inflammation, however, with such a state of nervous irritation and depression, and of the still more frequent transition of the one into the other, no one will doubt; and, indeed, this is abundantly admitted by the author himself.

"I have remarked that puerperal fever, if it do not rapidly prove fatal, generally merges into an inflammatory affection. The inflammation may attack the peritoneum, the ovaria, the uterus, or the intestines, all at once, successively, or be confined to one organ." (P. 16.)

That, in cases like the above, active antiphlogistic measures are indicated, is as certain as they were contraindicated in the cases previously referred to; and the sole merit of the author of the present work, as we have already said, consists in calling the attention of those who are unacquainted with it to the important fact. The following extract well illustrates the mistakes likely to be committed by a young and book-taught practitioner, and points out their fatal consequences.

"The various doctrines prevalent as to the nature and treatment of puerperal fever have been of this exclusive character. The opinions of their several promulgators are entitled to our respect, some almost to our adoption; but their indiscriminate application to every case named or misnamed puerperal fever cannot be too strongly reprobated. To illustrate the mode of their pernicious influence:—A female is attacked with genuine puerperal fever, and is visited by an exclusive disciple of the antiphlogistic doctrine. Believing, as his preconceived notions lead him to believe, that this fell inroad of disease can but in one way (the large abstraction of blood,) be successfully opposed, he plunges his lancet into the veins of his patient, prolongs the copious stream, and secures for her an almost certain passage into a better world. Another lying-in woman, by dint of good constitutional powers, we will suppose to have survived the essential stage of the malady, and to be attacked with *successive* inflammation. Her attendant may happen to be a disciple of the Hamiltonian school, who, under the impression that her complaint is genuine puerperal fever, alias *debility,* (that bugbear of practical medicine,) because forsooth she is in a puerperal condition, pours in his cordials to support a life he thus unconsciously destroys. Had this latter female happened to be seen by the former-mentioned practitioner, her life would have been in all probability saved; not from the one holding more correct views of the complaint's pathology than the other, but from the accidental *applicability* of those views to the *existing* state of the case. Again, imagine a woman attacked with a mild degree of puerperal fever: a disciple of Brenan visits her, prescribes turpentine, and on his next visit perceives the complaint removed. Exposing herself to cold two or three days afterwards, we will presume her seized with acute enteritis: her medical attendant sees her again, and, *because she is in the puerperal state and he entertains certain notions,* turpentine is again solely trusted to. To detail the consequences would be superfluous,—they are too easily inferred. Finally, to instance one more effect of these *exclusive* doctrines, an individual is attacked with undoubted puerperal fever: her attendant, not knowing what to do, (for, provided he be inexperienced, to what source of direction can he with confidence look?) equally fearing to stimulate or to deplete, orders perhaps a mild aperient and an evening sudorific draught. What is the result? The disease advances—the practitioner remains undecided—the friends are distressed—the woman dies! This is no ideal picture." (P. 17.)

A great desideratum in pathological and practical medicine still remains to be supplied,—namely, a full and accurate account, and consequent discrimination, of the various febrile and inflammatory affections which occur in the puerperal state; and which have so long been confounded under the general name of Puerperal Fever. Many materials for such a treatise exist in the numerous works on childbed diseases in many modern languages; but the construction from these of one connected whole, such as is now needed, requires not merely industry and learning and a mind disciplined in general logic and philosophy, but a long, extensive, and chastened personal experience of the very diseases in question. Why do not some of the metropolitan physicians, connected with the lying-in establishments of London or Dublin, devote themselves to this great task? We think we could point out more than one eminently qualified for it.

There is nothing peculiar in Dr. Alexander's treatment; nothing, indeed, that may not be found in every elementary work on the practice of medicine. Fomentations to the abdomen, enemata, bloodletting, calomel combined with Dover's powder, purgatives of spirits of turpentine and castor-oil, are the remedies recommended, according to the particular circumstances of the individual case; and we discover no precepts regulating their administration which are entitled to the praise of novelty, or even of particular clearness of enunciation. On the other hand, we find not a little that is vague, and some things rather contradictory.

The quotations which we have given from Dr. A.'s work sufficiently exhibit the inaccuracy and imperfections of his style, although many more unfavorable specimens might have been extracted; and we are sorry to say that here, as in most cases, inaccuracy of language is combined with incorrectness of argument and inconsequential ratiocination. We trust that, when we next meet Dr. Alexander in the field of literature, he will have learned to correct these deficiencies, which, however countenanced by the general carelessness of medical writers, are not the less discreditable to the members of a learned profession, and are most injurious to the philosophical character of medicine as a branch of general science.

